# Exercise‐induced changes in left ventricular strain are affected by interleukin‐6 activity: An exploratory analysis of a randomised‐controlled trial in humans with abdominal obesity

**DOI:** 10.1113/EP091800

**Published:** 2024-05-27

**Authors:** Simon Jønck, Mathilde Løk, Cody Durrer, Anne‐Sophie Wedell‐Neergaard, Louise Lang Lehrskov, Grit Elster Legaard, Rikke Krogh‐Madsen, Jaya Rosenmeier, Morten Asp Vonsild Lund, Bente Klarlund Pedersen, Helga Ellingsgaard, Ronan M. G. Berg, Regitse Højgaard Christensen

**Affiliations:** ^1^ Centre for Physical Activity Research Copenhagen University Hospital ‐ Rigshospitalet Copenhagen Denmark; ^2^ Department of Cardiology Copenhagen University Hospital ‐ Rigshospitalet Copenhagen Denmark; ^3^ Department of Biomedical Sciences, Faculty of Health and Medical Sciences University of Copenhagen Copenhagen Denmark; ^4^ Department of Dermatology and Allergy Copenhagen University Hospital ‐ Herlev and Gentofte Copenhagen Denmark; ^5^ Department of Oncology Copenhagen University Hospital – Herlev and Gentofte Copenhagen Denmark; ^6^ Department of Infectious Diseases Copenhagen University Hospital ‐ Hvidovre Copenhagen Denmark; ^7^ Department of Clinical Physiology and Nuclear Medicine Copenhagen University Hospital – Rigshospitalet Copenhagen Denmark; ^8^ Neurovascular Research Laboratory, Faculty of Life Sciences and Education University of South Wales Pontypridd UK

**Keywords:** cardiac adaptations, exercise, interleukin‐6

## Abstract

Whilst the exercise‐induced myokine interleukin‐6 (IL‐6) plays a beneficial role in cardiac structural adaptations, its influence on exercise‐induced functional cardiac outcomes remains unknown. We hypothesised that IL‐6 activity is required for exercise‐induced improvements in left ventricular global longitudinal strain (LV GLS). In an exploratory study 52 individuals with abdominal obesity were randomised to 12 weeks’ high‐intensity exercise or no exercise in combination with IL‐6 receptor inhibition (IL‐6i) or placebo. LV strain and volume measurements were assessed by cardiac magnetic resonance. Exercise improved LV GLS by −5.4% [95% CI: −9.1% to −1.6%] (*P* = 0.007). Comparing the change from baseline in LV GLS in the exercise + placebo group (−4.8% [95% CI: −7.4% to −2.2%]; *P *< 0.0004) to the exercise + IL‐6i group (−1.1% [95% CI: −3.8% to 1.6%]; *P* = 0.42), the exercise + placebo group changed −3.7% [95% CI: −7.4% to −0.02%] (*P* = 0.049) more than the exercise + IL6i group. However, the interaction effect between exercise and IL‐6i was insignificant (4.5% [95% CI: −0.8% to 9.9%]; *P* = 0.09). Similarly, the exercise + placebo group improved LV global circumferential strain by −3.1% [95% CI: −6.0% to −0.1%] (*P* = 0.04) more compared to the exercise + IL‐6i group, yet we found an insignificant interaction between exercise and IL‐6i (4.2% [95% CI: −1.8% to 10.3%]; *P* = 0.16). There was no effect of IL‐6i on exercise‐induced changes to volume rates. This study underscores the importance of IL‐6 in improving LV GLS in individuals with abdominal obesity suggesting a role for IL‐6 in cardiac functional exercise adaptations.

## Abstract (highlights)

- **What is the central question of this study?**
  Interleukin‐6 (IL‐6) is a myokine that is released from skeletal muscle following exercise and is involved in structural cardiac adaptations to exercise: is IL‐6 activity also involved in functional cardiac exercise adaptations?
- **What is the main finding?**
  In this randomised placebo‐controlled clinical trial in individuals with abdominal obesity, inhibition of IL‐6, relative to placebo, attenuated exercise‐induced left ventricular global longitudinal strain improvements measured by magnetic resonance. The findings indicate that IL‐6 may be involved in exercise‐induced improvements in cardiac function.

## 1 INTRODUCTION

Obesity is associated with cardiac morbidity and mortality and is an individual predictor of cardiac diastolic and systolic dysfunction, including impairments in left ventricular (LV) global longitudinal strain (GLS) (Blomstrand et al., 2018). Despite evidence that exercise is associated with fat loss and improvements in both cardiac structure and function, the underlying molecular mechanisms for cardiac adaptations to exercise training in individuals at risk for cardiac deterioration remains uncertain (Fernandes et al., 2015; Pinckard et al., 2019).

During exercise, skeletal muscle releases myokines into the circulation where they exert autocrine, paracrine and endocrine effects (Ostrowski et al., 1998; Pedersen, 2009). Interleukin‐6 (IL‐6) is the most extensively studied myokine and displays the earliest and most significant elevations in plasma concentrations (Hojman et al., 2019; Petersen & Pedersen, 2005). In a recent study in untrained men and women with abdominal obesity, inhibiting IL‐6 activity (using the IL‐6 receptor antibody tocilizumab) attenuated the increase in LV mass observed after a 12‐week high‐intensity interval training (HIIT) intervention (Christensen, Lehrskov, et al., 2019). This study thus indicated that IL‐6 is involved in exercise‐induced cardiac hypertrophy, which involves a balanced increase in cardiac volume and mass with a concomitant improvement in systolic function and improved or sustained diastolic function (Martinez et al., 2021; Shimizu & Minamino, 2016). Apart from the increase in LV mass, it is unknown whether IL‐6 is also involved in functional cardiac adaptations to exercise training. The existence of such a link is supported by the involvement of IL‐6 in regulating changes in response to exercise training in mass of visceral and epicardial adipose tissue (Christensen, Lehrskov, et al., 2019), both of which are fat depots associated with reduced systolic and diastolic dysfunction (Christensen, Hansen, et al., 2019; Liu et al., 2023). Epicardial adipose tissue (EAT) has in particular, due to its anatomical proximity and metabolic hyperactivity, been proposed to impact cardiac function by secreting inflammatory factors and applying mechanical stress on the myocardium (Christensen, Hansen, et al., 2019).

In this exploratory study we aimed to investigate whether the previously observed IL‐6‐dependent changes in epicardial adipose tissue mass and LV mass (Christensen, Lehrskov, et al., 2019), induced by a 12‐week HIIT regimen, were accompanied by IL‐6‐dependent changes in cardiac function. Cardiac systolic function was measured by change in myocardial deformation through LV strain measurements, and LV systolic and diastolic volume rates normalised to LV end‐diastolic volume (LV EDV) to measure normalised peak ejection rate (NPER) and normalised peak filling rate (NPFR) (Cuocolo et al., 1990). All measurements were obtained by cardiac magnetic resonance imaging (CMR) (Kermer et al., 2020; Vos et al., 2022). The hypothesis of this study was that IL‐6 receptor inhibition (IL‐6i) would attenuate a 12‐week HIIT‐induced improvement in LV systolic function assessed by LV GLS.

## 2 METHODS

### 2.1 Ethical approval

Prior to commencement, the study was granted approval from The Danish Capital Region Ethics Committee (H‐16018062) and registered on clinicaltrials.gov (NCT02901496). The study adhered to the principles outlined in the *Declaration of Helsinki*. All study participants provided written informed consent prior to participation. The study was an exploratory analysis on data from a 12‐week, double‐blinded, randomised, placebo‐controlled, drug and exercise training study, which took place at the Centre for Physical Activity Research (CFAS) at Rigshospitalet, Denmark from August 2016 to April 2018 (Wedell‐Neergaard et al., 2019).

The study protocol has been published (Christensen et al., 2018). In brief, individuals were eligible if they were physically inactive and abdominally obese. This obesity criterion was met through a waist‐to‐height ratio ≥0.5 and/or waist circumference ≥88 cm for women and ≥102 cm for men.

**FIGURE 1:**
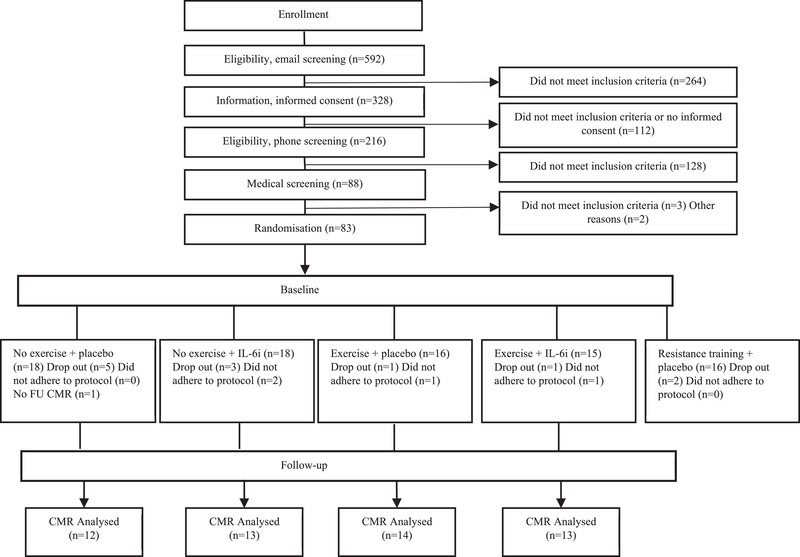
Flowchart of study. This figure is modified, with permission, from a previous publication (Wedell‐Neergaard et al., 2019). Importantly, the resistance training group is not part of this study but was published separately (Christensen, Wedell‐Neergaard, et al., 2019). CMR, cardiac magnetic resonance imaging; FU, follow‐up; IL‐6i, interleukin‐6 receptor inhibition.

The allocation of eligible participants into five distinct groups was achieved through computer‐generated block randomisation with a 1:1:1:1:1 ratio. Given the mechanistic nature of the study, participants deviating from the protocol were excluded (adequate adherence was defined as ≥80% attendance to the exercise training intervention and complete adherence to IL‐6i/placebo intervention.) The five randomised groups were (1) no exercise + placebo, (2) no exercise + IL‐6i, (3) exercise + placebo, (4) exercise + IL‐6i, and (5) resistance exercise + placebo. Notably and as specified in the protocol, specific groups were allocated to different secondary/exploratory analyses. Thus, the resistance exercise + placebo group was not part of the present study (Figure 1).

### 2.2 HIIT intervention

The exercise + placebo and the exercise + IL‐6i groups performed HIIT on a bike ergometer (TehcnoGym, Pedan A/S, Copenhagen, Denmark) for 45 min, 3 times weekly for 12 weeks. The high intensity increased progressively from 75% to 85% maximal oxygen uptake (${\dot{V}}_{O_{2}\max}$, mL/min) during the study period. All sessions were supervised and took place at CFAS. A detailed description of the intervention has previously been described (Christensen et al., 2018).

### 2.3 IL‐6 receptor inhibition

Participants assigned to IL‐6i (tocilizumab, a recombinant humanised monoclonal IL‐6 receptor antibody at a concentration of 20 mg/mL from Roche Pharma AG, Basel, Switzerland) were administered infusions at the onset of the intervention and subsequently every fourth week throughout the 12‐week intervention, following the approved doses of 8 mg/kg body weight, or a maximum dose of 800 mg (RoActemra, European Medicines Agency: https://www.ema.europa.eu/en/medicines/human/EPAR/roactemra). Those randomised to the placebo group received saline infusions.

### 2.4 Maximal oxygen uptake

${\dot{V}}_{O_{2}\max}$ (mL/min) was determined during a bicycle ergometer test (Monark Ltd, Varberg, Sweden). The participants first warmed up for 5 min at 70 W, and warm‐up was followed by a systematic 15 W increase per minute until exhaustion. Breath‐by‐breath measurements of ${\dot{V}}_{O_{2}}$ was determined by indirect calorimetry (Quark b2, CosMed, Rome, Italy).

### 2.5 Cardiac magnetic resonance imaging

CMR was conducted using a 1.5 T whole‐body MRI scanner (Ingenia; Philips, Eindhoven, Holland), acquiring steady‐state free precession cine images with typical parameters. The full CMR protocol has been described in detail previously (Christensen, Wedell‐Neergaard, et al., 2019). Semiautomatic calculations were performed using dedicated analysis software (CVI42®, version 5.11; Circle Cardiovascular Imaging, Calgary, AB, Canada), applying endocardial and epicardial contours for strain analysis. According to guidelines manual, contour corrections were performed if necessary (Schulz‐Menger et al., 2020), LV trabeculae were included in cavity volume, and papillary muscles were excluded from cavity volume calculations (Weinsaft et al., 2008). NPER (LV EDV/s) and NPFR (LV EDV/s) correspond to the maximal change in LV volume between sequential temporal phases during systole and diastole, normalised to LV end‐diastolic volume (EDV), respectively. LV GLS, LV global circumferential strain (LV GCS), and LV global radial strain short axis (LV GRS_sax_) are measures of myocardial shortening across different segments (Zhang et al., 2019). LV GLS and LV GCS have negative values, indicating improved cardiac function with decreased strain percentage (smaller value). LV GRS_sax_ has positive values, indicating improved cardiac function with increased strain percentage (larger value). In accordance with age‐matched healthy controls, using CVI42 and not stratifying for sex, the normal 1.5 T two‐dimensional CMR‐based range (%) for LV GLS, LV GCS and LV GRS_sax_ was assumed to be −21.2 to −12.8, −24.2 to −15.0 and 17.5 to 40.7, respectively (Lim et al., 2021). To assess cardiac function, we determined the difference in volume rates and strain measurements in the LV before and after 12 weeks of exercise training. All analyses were performed in a blinded manner.

### 2.6 Statistical analysis

All statistical analyses were conducted using R (version 4.3.0) (R Core Team, 2022). All continuous outcomes were analysed using a constrained baseline mixed effects model (Bates et al., 2015). The model included fixed effects for time point (baseline vs. follow‐up), sex (male vs. female), exercise (coded 0 if time point = baseline or group = 1 or 2; and coded 1 otherwise) and IL‐6i (coded 0 if time point = baseline or group = 1 or 3; and coded 1 otherwise) an interaction term for exercise × IL‐6i, and random intercepts for participant. The interaction exercise × IL‐6i represents the extent to which IL‐6i moderates the effect of exercise on the outcome of interest. Model assumptions were checked via visual inspection of fitted values versus residuals plot and normal probability plots. Estimated marginal means and contrasts were calculated (Lenth, 2023). If warranted, outcomes were log‐transformed to satisfy model assumptions; in these cases, estimated marginal means were back‐transformed to their original scale and contrasts are presented as percentage differences (i.e., not in their original scale). To test for any main effects of exercise, IL‐6i, or exercise × IL‐6i interaction, a type III ANOVA was conducted (Fox et al., 2023). Data are presented as means ± SD for baseline, and follow‐up data are presented as estimated marginal means [95% CI]. Due to the exploratory nature of this secondary analysis, no default adjustments for multiplicity were performed. A two‐sided *P *< 0.05 was considered statistically significant.

## 3 RESULTS

A total of 67 participants were randomised. Ten participants withdrew, and four participants were excluded as they did not adhere to the predefined per‐protocol criteria; two of them were in the no exercise + IL‐6i, one in the exercise + placebo and one in the exercise + IL‐6i group. One participant in the no exercise + placebo group did not complete the follow‐up CMR scan. Thus, a total of 52 participants were included in the analysis (Figure 1). Baseline characteristics are reported in Table 1; briefly the mean age was 44 (SD 13) years, 13 (25%) were males, and mean BMI was 33 (SD 5) kg/m^2^. Baseline strain values were all within the normal range (Kawel‐Boehm et al., 2020; Lim et al., 2021). There were no apparent significant differences in all baseline values between the groups. Daily free‐living physical activity and self‐reported food intake did not differ between groups, along with adverse events; these results are reported elsewhere (Wedell‐Neergaard et al., 2019). It has been previously reported that basal plasma levels of IL‐6 and the IL‐6 soluble receptor did not change by exercise, and efficacy of the intervention was ensured by noting that high‐sensitivity‐CRP was suppressed in the groups receiving IL‐6i, which was expected since IL‐6 is the main inducer of CRP (Wedell‐Neergaard et al., 2019).

**TABLE 1:** Baseline characteristics.

	No exercise + placebo (*n* = 12)	No exercise + IL‐6i (*n* = 13)	Exercise + placebo (*n* = 14)	Exercise + IL‐6i (*n* = 13)	Overall (*n* = 52)
Anthropometric data					
Female/male (*n*)	10/2	8/5	11/3	10/3	39/13
Age (years)	47 (12)	44 (12)	39 (14)	44 (12)	44 (13)
Weight (kg)	97 (18)	99 (17)	92 (14)	96 (15)	95 (16)
Waist (cm)	108 (12)	110 (15)	104 (14)	106 (11)	107 (13)
Waist‐to‐height ratio	0.63 (0.08)	0.64 (0.08)	0.62 (0.08)	0.62 (0.06)	0.63 (0.07)
Body mass index (kg/m^2^)	34(6)	33 (5)	33 (5)	33 (5)	33 (5)
Systolic BP (mmHg)	124 (20)	126 (18)	128 (21)	134 (20)	129 (19)
Diastolic BP (mmHg)	84 (9)	86 (13)	87 (10)	89 (9)	87 (10)
Resting HR (beats/min)	71 (13)	73 (15)	68 (7)	73 (11)	72 (12)
Plasma IL‐6 (pg/mL)	0.8 (0.5)	1.0 (0.9)	0.7 (0.3)	0.7 (0.4)	0.8 (0.7)
Cardiac measures					
LV GLS (%)	−16.2 (1.7)	−15.5 (1.7)	−15.3 (1.3)	−16.1 (2.1)	−15.7 (1.7)
LV GCS (%)	−17.9 (1.6)	−16.9 (1.6)	−17.5 (1.2)	−17.2 (1.9)	−17.4 (1.6)
LV GRS_sax_ (%)	30.8 (4.4)	28.3 (3.3)	29.5 (2.6)	28.8 (4.9)	29.3 (3.9)
NPER (LV EDV/s)	3.6 (0.5)	3.7 (0.7)	3.4 (0.4)	3.5 (0.7)	3.5 (0.6)
NPFR (LV EDV/s)	3.3 (0.6)	3.3 (0.6)	3.4 (0.7)	3.4 (0.7)	3.3 (0.6)
Fitness measures^a^					
Relative ${\dot{V}}_{O_{2}\max}$ (mL/min/kg)	27.96 (6)	26.34 (5)	31.07 (5)	30.79 (6)	29.04 (6)

*Note*: Values are presented as mean (SD).

^a^ Missing value (*n* = 1) in the No exercise + placebo group. Part of this table has been previously published (Christensen, Wedell‐Neergaard, et al., 2019). Abbreviations: BP, blood pressure; GCS, global circumferential strain; GLS, global longitudinal strain; GRS, global radial strain; HR, heart rate; IL‐6, interleukin‐6; IL‐6i, interleukin‐6 receptor inhibition; LV, left ventricle; LV EDV, left ventricle end‐diastolic volume; NPER, peak ejection rate normalised to LV EDV; NPFR, peak filling rate normalised to LV EDV; ${\dot{V}}_{O_{2}}$, oxygen consumption.

### 3.1 Strain modalities

**FIGURE 2:**
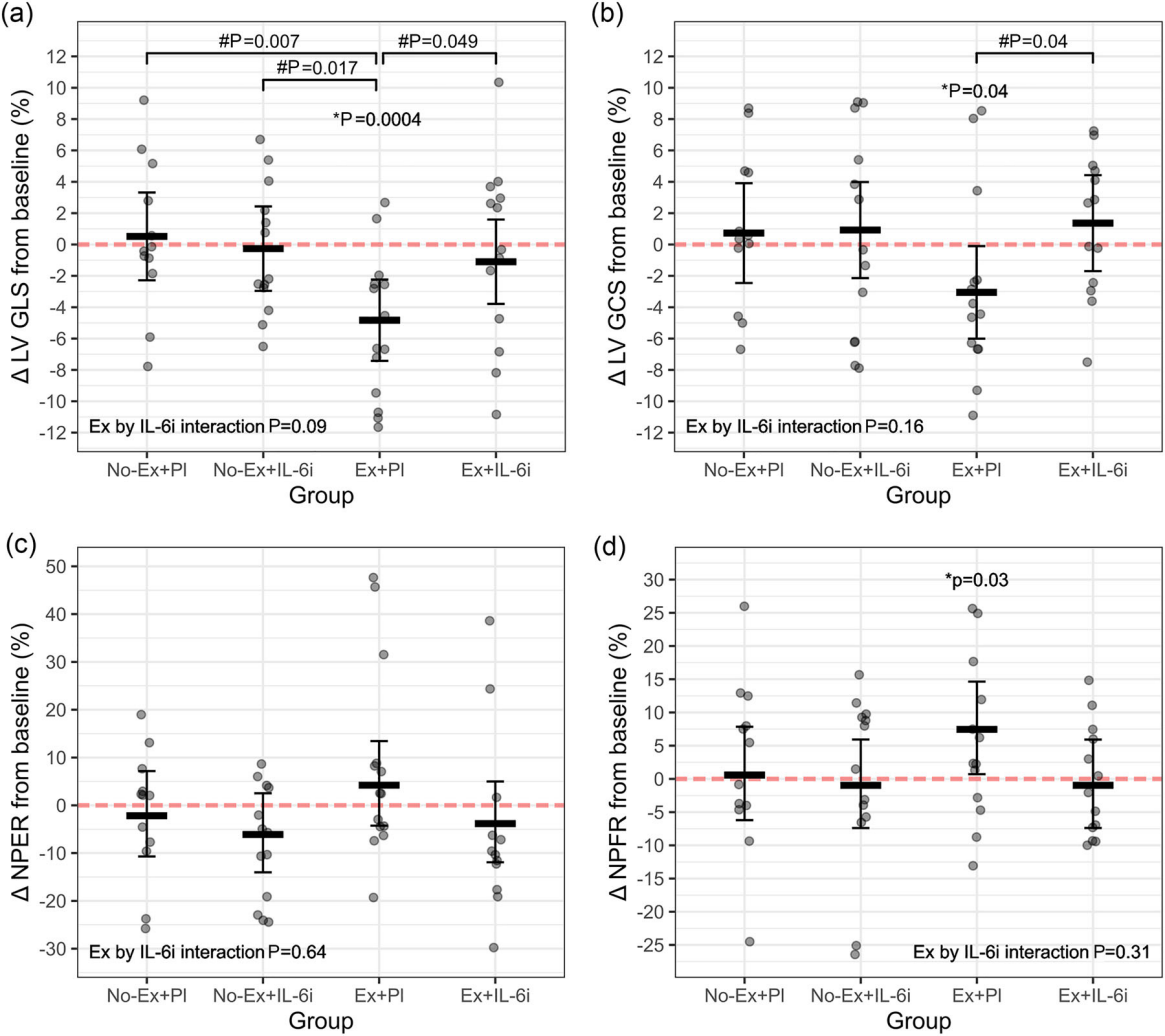
Effects of exercise on left ventricular function in combination with placebo or IL‐6i (*n* = 52). (a) LV GLS relative (%) change from baseline; (b) LV GCS relative (%) change from baseline; (c) NPER relative (%) change from baseline; (d) NPFR relative (%) change from baseline. Significant differences are denoted: *within‐group difference from baseline; #between‐group difference in change from baseline (*P *< 0.05). Ex, exercise; IL‐6i; interleukin‐6 receptor inhibition; LV, left ventricle; LV GCS, LV global circumferential strain; LV GLS, LV global longitudinal strain; NPER, normalised peak ejection rate; NPFR, normalised peak filling rate; Pl, placebo.

Exercise training demonstrated a significant main effect on LV global longitudinal strain (GLS), revealing an improvement of −5.4% [95% CI: −9.1% to −1.6%] (*P* = 0.007) (Table 2 and Figure 2a). LV GLS changed −4.8% [−7.4% to −2.2%] (*P* = 0.0004) in the exercise + placebo group and −1.1% [−3.8% to 1.6%] (*P* = 0.42) in the exercise + IL‐6i group (Figure 2a). LV GLS showed a −3.7% [−0.02% to −7.4%] greater change in the exercise + placebo than in the exercise + IL‐6i group (*P* = 0.049) (Figure 2a). However, there was not a significant interaction between IL‐6i and exercise on changes in LV GLS (4.5 % [−0.8% to 9.9%]; *P* = 0.097). LV GLS remained unaffected in the no exercise + placebo group (0.5 [95% CI: −2.3% to 3.3%]; *P* = 0.71) and in the no exercise + IL‐6i group (−0.3 [95% CI: −3.0% to 2.4%]; *P* = 0.84) (Table 2 and Figure 2a). There was no difference in change between the two non‐exercising groups (*P* = 0.69) (Figure 2a).

**TABLE 2:** Within group changes and main effects of exercise, IL‐6i and their interaction.

	No exercise + placebo [95% CI] (*n* = 12)	No exercise + IL‐6i [95% CI] (*n* = 13)	Exercise + placebo [95% CI] (*n* = 14)	Exercise + IL‐6i [95% CI] (*n* = 13)	Main effect of exercise [95% CI] (*P*)	Main effect of IL‐6i [95% CI] (*P*)	Interaction IL‐6i and exercise [95% CI] (*P*)
LV GLS							
Follow‐up means (12 weeks) (%)	−15.2 [−15.8 to −14.6]	−15.3 [−15.9 to −14.7]	−16.0 [−16.6 to −15.4]	−15.4 [−16.51 to −14.8]	—	—	—
Relative change (%) from baseline	0.5 [−2.3 to 3.3]	−0.3 [−3.0 to 2.4]	−4.8 [−7.4 to −2.2]	−1.10 [−3.8 to 1.6]	−5.4 [−9.1 to −1.6] (0.007)	−0.8 [−4.6 to 3.1] (0.69)	4.51 [−0.8 to 9.9] (0.09)
LV GCS							
Follow‐up means (12 weeks) (%)	−16.9 [−17.6 to −16.2]	−16.8 [−17.5 to −16.2]	−17.5 [−18.2 to −16.9]	−16.8 [−17.4 to −16.1]	—	—	—
Relative change (%) from baseline	0.7 [−2.5 to 3.9]	0.9 [−2.1 to 4.0]	−3.1 [−6.0 to −0.1]	1.4 [−1.7 to 4.4]	−3.8 [−8.1 to 0.5] (0.08)	0.2 [−4.2 to 4.6] (0.93)	4.2 [−1.8 to 10.3] (0.17)
LV GRS_sax_							
Follow‐up means (12 weeks) (%)	28.6 [26.8 to 30.4]	29.1 [27.4to 30.8]	29.4 [27.7 to 31.1]	28.4 [26.7to 30.2]	—	—	—
Relative change (%) from baseline	0.1 [−4.8 to 5.1]	2.0 [−2.8 to 6.8]	2.9 [−1.7 to 7.5]	−0.4 [−5.2 to 4.4]	2.8 [−3.9 to 9.5] (0.41)	1.9 [−4.9 to 8.2] (0.58)	−5.2 [−14.6 to 4.3] (0.28)
NPER							
Follow‐up means (12 weeks) (LV EDV/s)	3.32 [3.0 to 3.7]	3.2 [3.0 to 3.5]	3.6 [3.3 to 3.9]	3.3 [3.0 to 3.6]	—	—	—
Relative change (%) from baseline	−3.7 [−12.4 to 5.9]	−6.2 [−14.1 to 2.4]	4.8 [−4.3 to 13.4]	−3.9 [−11.9 to 4.9]	8.2 [−4.4 to 22.3] (0.21)	−2.7 [−14.2 to 10.4] (0.67)	−5.2 [−20.2 to 12.6] (0.54)
NPFR							
Follow‐up means (12 weeks) (LV EDV/s)	3.2 [2.9 to 3.5]	3.2 [2.9 to 3.4]	3.4 [3.2 o 3.7]	3.2 [2.9 to 3.4]	—	—	—
Relative change (%) from baseline	−1.0 [−7.8 to 6.3]	−1.1 [−7.4 to 5.6]	7.3 [0.7 to 14.3]	−1.1 [−7.3 to 5.7]	8.4 [−1.3 to 19.1] (0.09)	−0.1 [−9.3 to 9.9] (0.97)	−7.7 [−19.1 to 5.3] (0.23)

*Note*: Values are presented as estimated marginal means (means adjusted for baseline) with [95% CI]. A type III ANOVA tested the main effects of IL‐6i, exercise, and IL‐6i * exercise interaction. Abbreviations: IL‐6i, interleukin‐6 inhibition; LV, left ventricle; LV EDV, LV end‐diastolic volume; LV GCS, LV global circumferential strain; LV GLS, LV global longitudinal strain; LV GRS, LV global radial strain; NPER, peak ejection rate normalised to LV EDV; NPFR, peak filling rate normalised to LV EDV.

In contrast to LV GLS, there was no significant main effect of exercise on LV GCS (−3.8% [−8.1% to 0.5%]; *P* = 0.09) and the interaction between IL‐6i and exercise was not significant (4.2% [95% CI: −1.8% to 10.3%]; *P* = 0.16) (Table 2 and Figure 2b). However, there was a significant within‐group change in the exercise + placebo group of −3.1% [−6.0% to −0.1%] (*P* = 0.04), which was not observed in the exercise + IL‐6i group (1.4% [−1.7% to 4.4%]; *P* = 0.37) (Figure 2b). Consequently, GCS showed a 4.4% [0.2% to 8.6 %] (*P* = 0.04) greater change in the exercise + placebo than in the exercise + IL‐6i group (*P* = 0.039) (Figure 2b). LV GCS remained unchanged from baseline in the no exercise + placebo group (0.7% [95% CI: −2.5% to 3.9%]; *P* = 0.65) and in the no exercise + IL‐6i group (0.9% [95% CI: −2.1% to 4.0%]; *P* = 0.55) (Table 2 and Figure 2b). There was no difference in change between the two non‐exercising groups (*P* = 0.93) (Figure 2b). LV GRS_sax_ remained unchanged in all groups (Table 2).

### 3.2 Systolic and diastolic volume rates

Exercise training had no significant main effect on change on the systolic volume rate (NPER) (8.2% [95% CI: −4.4% to 22.3%]; *P* = 0.21), and no significant interaction was observed between exercise and IL‐6i (−3.9% [95% CI: −18.9% to 14.0%]; *P* = 0.65) (Table 2 and Figure 2c). The within‐group NPER remained unchanged from baseline in both the exercise + placebo group (4.8% [95% CI: −4.3% to 13.4%]; *P* = 0.33) and the exercise + IL‐6i group −3.9% [95% CI: −11.9% to 4.9%] (*P* = 0.37) (Table 2), with no significant difference in the between‐group analysis (*P* = 0.17). In the no‐exercise groups, the within‐group changes to NPER were not significant both with placebo (−3.7% [95% CI: −12.4% to 5.9%]; *P* = 0.43) and with IL‐6i (−6.2% [95% CI: −14.1% to 2.9%]; *P* = 0.15) (Table 2), with no difference in change when comparing the groups (*P* = 0.67).

Similar to NPER, the main effect of exercise did not significantly improve the diastolic volume rate (NPFR) (8.4% [95% CI: −1.3% to 19.1%]; *P* = 0.09), with a non‐significant interaction effect between exercise and IL‐6i (−6.4% [95% CI: −18.0% to 6.9%]; *P* = 0.32) (Table 2 and Figure 2d). The exercise + placebo group displayed a significant within‐group change (7.3% [95% CI: 0.7% to 14.3%]; *P* = 0.03), whereas the exercise + IL‐6i group did not (−1.1% [95% CI: −7.3% to 5.7%]; *P* = 0.75) (Table 2); however the between‐group difference did not to show significance (7.9% [95% CI: −0.8% to 15.7%]; *P* = 0.07). We found no change from baseline in NPFR in both the no exercise + placebo group (−1.0% [95% CI: −7.8% to 6.3%]; *P* = 0.77) and the no exercise + IL‐6i group (−1.1% [95% CI: −7.4% to 5.6%]; *P* = 0.73) (Table 2) with no difference in change when comparing the groups (*P* = 0.98).

### 3.3 Epicardial adipose tissue and LV functional measures

As previously published (Christensen, Lehrskov, et al., 2019) EAT mass was reduced by 8 g [95% CI: 2–14 g] (*P* *= *0.004) more when the change in exercise + placebo was compared to no exercise + placebo. In contrast, EAT mass remained unchanged following exercise + IL‐6i compared to no exercise + IL‐6i (1 g [95% CI: −5 to 7 g]; *P = *0.74). The difference in EAT change between the exercise + placebo group and the exercise + IL‐6i group was 9 g (95% CI: 4–15 g], *P* = 0.002). The change in EAT was inversely associated with changes in NPFR (−0.016% [95% CI: −0.031% to 0.00%]; *P* = 0.047), but not with other LV functional measures. See Supporting Information for the complete raw dataset.

## 4 DISCUSSION

This study showed that IL‐6i attenuates improvements in LV GLS following a 12‐week HIIT intervention, indicating that IL‐6 activity plays a role in exercise‐induced improvements in LV GLS. In the no‐exercise groups, we did not find changes in cardiac function, irrespective of IL‐6i, which points to an exercise‐specific role of IL‐6 activity for functional cardiac adaptations. To our knowledge, this is the first study that has analysed the role of IL‐6 activity in cardiac strain adaptations to exercise.

### 4.1 Cardiac functional adaptations to exercise

Long term exercise leads to cardiac adaptations in humans including increase in cardiac myocyte size, that is, cardiac physiological hypertrophy, whilst maintaining or improving contractile function, for example, global longitudinal strain, LV GLS (Lavie et al., 2015; Nystoriak & Bhatnagar, 2018). The main effect of exercise in our study improved LV GLS by −5.4%, which aligned with a −5.81% improvement in LV GLS in a 12‐week exercise study in a similar cohort with metabolic syndrome (Hollekim‐Strand et al., 2014), and also aligned with a systematic review summarising improvements in LV GLS with exercise interventions of varying intensity and duration in cardiovascular disease populations (Murray et al., 2022). We also found an improvement in LV GCS in the exercise + placebo group, which aligns with some studies (Scharf et al., 2015) whilst other studies did not find changes in LV GCS following exercise training (Engvall et al., 2021). Collectively, our findings confirm that exercise improves LV GLS and GCS, but the effect of exercise on circumferential strain is not yet fully clarified.

### 4.2 IL‐6 plays a role for exercise‐induced cardiac strain adaptations

In contrast to the changes in LV GLS in the exercise + placebo group, LV GLS did not change following exercise in the group that received IL‐6i concomitantly. Our study, therefore, suggests the involvement of IL‐6 in exercise‐induced improvements in cardiac systolic function, specifically in LV GLS. A significant difference in the change of LV GLS was observed between exercise + placebo and exercise + IL‐6i. Although IL‐6i appears to modify the effect of exercise on LV GLS, the interaction effect did not reach statistical significance (*P* = 0.09). Given the group distinctions, the non‐significant interaction effect might be attributed to statistical power limitations. Whilst the impact of IL‐6 activity on systolic function is biologically plausible, the current investigation does not definitively establish whether alterations in LV GLS are directly influenced by IL‐6 or whether they result from indirect mechanisms related to metabolic shifts mediated by IL‐6. For example, given that epicardial fat is associated with cardiac systolic and diastolic dysfunction and IL‐6 is necessary for exercise to reduce epicardial adipose tissue mass, it is possible that exercise‐induced IL‐6, by lowering epicardial fat mass and fat inflammation, becomes cardioprotective (Christensen, Hansen, et al., 2019, 2019; Packer, 2018; Serrano‐Ferrer et al., 2016). We observed an association of the changes in epicardial adipose tissue mass and NPFR, which indicates that epicardial adipose tissue reduction is associated with improved/increased NPFR. However, the change in epicardial adipose tissue mass was not associated with other changes of LV function, which highlights that the effects of exercise‐induced IL‐6 on the myocardium may also be through other mechanisms.

The improvement in LV GLS by exercise training is suggested to be attributed, at least in part, to an increase in cardiomyocyte size (Bass‐Stringer et al., 2021) combined with an increase in numbers of functional sarcomere units (Hastings et al., 2023) and increased Ca^2+^ sensitivity in the myofilaments (Wisløff et al., 2001), which improves contractile function following exercise training. Whether IL‐6 activity plays a role on the myocardium is not fully understood, but IL‐6 is an anabolic molecule which has been shown to be essential for regulating skeletal muscle hypertrophy in mice following an exercise load (Serrano et al., 2008). This finding was extended to humans as it was demonstrated that IL‐6 activity plays a role in physiological LV mass expansion as a response to exercise (Christensen, Lehrskov, et al., 2019). Moreover, IL‐6 activity may also enhance myocardial contractility, since in vitro studies exploring the acute effects of IL‐6 on cardiomyocytes have demonstrated a role in protecting mitochondrial function through the phosphoinositide 3‐kinase pathway (Smart et al., 2006) and promoting positive contractility outcomes (Maxeiner et al., 2014). In vivo mouse studies also demonstrated an increase in IL‐6 receptor expression on the myocardium following exercise, rendering cardiomyocytes more responsive to IL‐6 (McGinnis et al., 2015). Other cardioprotective functions of acute IL‐6 activity include reductions in oxidative stress and apoptosis in cardiomyocytes and prevention of dilatation in response to pressure‐overload (Fontes et al., 2015). Overall, it is evident that the pleiotropic cytokine IL‐6 can acutely influence cardiomyocytes. Whilst many studies have explored the detrimental cardiovascular effects of chronic increased IL‐6 (Mooney et al., 2023; Mossmann et al., 2022), both in vitro and In vivo research in both mice and humans suggests a beneficial role in acute scenarios that is not fully understood, and our present study adds that IL‐6 plays a role in functional cardiac adaptations to exercise, namely improvements in LV GLS.

### 4.3 Exercise and IL‐6 effects on volume rates

In this study we did not observe effects of exercise or IL‐6 inhibition on changes in systolic volume rates. NPER was normalised to LV EDV, as peak filling rate is anticipated to rise with an exercise‐induced elevation in LV EDV (Izem et al., 2021). Given the exercise‐induced increase in LV GLS, this makes the absence of a significant main effect of exercise on NPER counterintuitive. This discrepancy may be explained by the fact that NPER is influenced by the LV ejection fraction (Cuocolo et al., 1990), a volume change that is less sensitive as an indicator of LV function changes compared to the measurement of mechanical changes in LV through strain (Smiseth et al., 2016). Hence, resting LV ejection fraction typically remains unaffected by exercise in healthy individuals (Pluim et al., 2000).

In terms of diastolic filling rate, NPFR exhibited an increase exclusively in the group that exercised, which indicates that the exercise‐induced improvement in LV GLS was accompanied by a concomitant improvement in diastolic function which is in accordance with the literature (Belardinelli et al, 1995). The mechanism by which exercise affects diastolic filling rate is complex and not fully understood, but markers that may increase diastolic function include loss of epicardial fat mass (Park et al., 2014), which also differed across the groups (Christensen, Lehrskov, et al., 2019) and was associated with improvement in NPFR in this study, suggesting that the significant within‐group change observed exclusively in the exercise + placebo group may be influenced by non‐myocardial factors. Given the small interaction effect between exercise and IL‐6i, both systolic and diastolic volume rate assessments indicated that the impact of IL‐6i seemed negligible. Considering the relatively modest sample size and high variance, the observed lack of statistical power could provide an explanation for the findings related to volume rates.

### 4.4 Limitations

This study has several limitations. First, the study results are exploratory, which limits the conclusions that can be drawn. Second, only participants adhering to the intervention were included, but due to its mechanistic purpose, the significance of a potential bias is limited. Third, the relatively small study size introduces susceptibility to type II errors, likely reflected by the lack of a significant interaction between exercise and IL‐6i on the effect of LV GLS. Fourth, we did not stratify for sex in our randomisation to groups and there may be a sex‐driven difference in IL‐6 secretion due to a larger skeletal muscle mass in men. However, research have found no sex‐driven differences in left ventricular adaptions after 3 months of endurance exercise (Howden et al., 2015). Fifth, the individual strain measures have different reproducibility, where the radial strain, GRS, exhibits lower reproducibility due to the diminished imaging resolution in the radial direction and thereby increases variability of the findings (Kawel‐Boehm et al., 2020). Finally, the cardiac adaptations induced by exercise are confined to the study duration (12 weeks), and the long‐term effects remain unknown.

In conclusion, this study demonstrates that a 12‐week HIIT‐based exercise intervention improves cardiac function in abdominal obese individuals. Of note, our findings suggest that exercise‐induced IL‐6 activity may play a role in the improvement in LV systolic function.

## AUTHOR CONTRIBUTIONS

Anne‐Sophie Wedell‐Neergaard, Louise Lang Lehrskov, Grit Elster Legaard, Rikke Krogh‐Madsen, Jaya Rosenmeier, Bente Klarlund Pedersen, Helga Ellingsgaard and Regitse Højgaard Christensen designed the study; Anne‐Sophie Wedell‐Neergaard, Louise Lang Lehrskov, Grit Elster Legaard, Regitse Højgaard Christensen, Simon Jønck, Mathilde Løk and Morten Asp Vonsild Lund collected the data; all authors interpreted the data; Cody Durrer and Simon Jønck performed the analyses; Simon Jønck and Regitse Højgaard Christensen wrote the first draft; all authors revised the final version of the manuscript. All authors have read and approved the final version of this manuscript and agree to be accountable for all aspects of the work in ensuring that questions related to the accuracy or integrity of any part of the work are appropriately investigated and resolved. All persons designated as authors qualify for authorship, and all those who qualify for authorship are listed.

## CONFLICT OF INTEREST

None declared.

## Supporting information

Raw dataset. The complete raw dataset, along with explanations of variables, can be accessed in the online version of this article.

## Data Availability

Data availability statement The data supporting the findings of this study are available in Supporting Information.
